# Observing super-coarse carbonaceous aerosol particles containing chloride in a tropical savanna climate at an agro-forest site in Thailand

**DOI:** 10.1007/s11356-024-35486-x

**Published:** 2024-11-06

**Authors:** Arika Bridhikitti, Chananphat Kumsawat, Nutthanaphat Phitakpinyo, Sirawich Sontisaka, Ratipong Naksaro, Weerachon Sawangproh, Tomoki Nakayama

**Affiliations:** 1 Environmental Engineering and Disaster Management Program, School of Interdisciplinary Studies, Mahidol University Kanchanaburi Campus, 199 Moo 9, Lumsum Sub-District, Saiyok District, Kanchanaburi, 71150 Thailand; 2 Mahidol-York Interdisciplinary Living Research Excellence Center, Mahidol University Kanchanaburi Campus, 199 Moo 9, Lumsum Sub-District, Saiyok District, Kanchanaburi, 71150 Thailand; 3 Conservation Biology Program, School of Interdisciplinary Studies, Mahidol University Kanchanaburi Campus, 199 Moo 9, Lumsum Sub-District, Saiyok District, Kanchanaburi, 71150 Thailand; 4 Faculty of Environmental Science, Nagasaki University, 1-14 Bunkyo-Machi, Nagasaki City, Nagasaki 8528521 Japan

**Keywords:** SEM/EDS; FTIR Carbonaceous aerosols, Biomass burning, Chloride aerosol, Coarse aerosol particles

## Abstract

Coarse aerosol particles containing chloride in tropical forests are significant for understanding biogeochemical cycles and atmospheric processes, with implications for environmental health and climate change mitigation. This study explored the sources of super-coarse carbonaceous aerosol particles containing chloride in a tropical savanna climate. Aerosol samples were collected from an agro-forest site in Thailand during the dry season and analyzed using scanning electron microscopy (SEM), energy-dispersive X-ray spectroscopy (EDS), and Fourier-transform infrared (FTIR) spectroscopy. By examining the morphology and elemental compositions of individual aerosol particles, along with employing Positive Matrix Factorization (PMF) and backward trajectory analysis, potential sources were identified. The findings revealed two primary sources for the super-coarse aerosol particles: a mixture of biomass burning smoke and inorganic salts (likely from saline soil and sea salt), as well as halophilic fungal spores. FTIR analysis indicated the presence of compounds linked to biomass burning and clay minerals, influenced by prevailing northeast and southeast winds. Recommendations for future research include continued monitoring, correlation with meteorological parameters, and the application of transmission electron microscopy (TEM) for more detailed visualization and confirmation of aerosol sources.

## Introduction

Globally, coarse (2.5–10 μm) and super-coarse (10–62.5 μm) dust aerosols comprise a substantial proportion, approximately 85%, of the total dust mass in the atmosphere (Adebiyi and Kok 2020). Despite their significant presence, recent climate models consistently overlook these coarse and super-coarse dust particles, leading to an underestimation of their impacts on various environmental components, including ocean ecosystems, land biogeochemistry, atmospheric chemistry, cloud formation, and global climate dynamics (Adebiyi and Kok 2020). The coarse-mode aerosol particles primarily originate from natural sources such as dust and sea salt, with their mixing state influenced by fine-mode aerosols (Koulouri et al. 2008). These larger aerosol particles, including coarse and super-coarse fractions, have been observed to undergo long-range transport, as demonstrated during events like the Asian Dust outbreak in 1986, where Aeolian particles were collected over distances exceeding 10,000 km from their source (Adebiyi and Kok 2020). Research conducted at a remote high alpine site in Switzerland found that the coarse aerosol fraction exhibited high concentrations of calcium and nitrate, suggesting a potential contribution from Saharan dust (Cozic et al. 2008). The transport of these coarse dust particles was found to have significant impacts on local topsoil nutrient depletion, primarily in the form of calcium nitrate, and regional air quality (Hand et al. 2017). In our study, we focused on individual aerosol particles collected at an agro-forest site in a tropical savanna climate in Thailand. Sampling was conducted during the dry biomass burning season in January 2022, revealing the consistent presence of super-coarse carbonaceous aerosol particles containing chloride in many samples.

Primary biological aerosol particles (PBAPs) are increasingly recognized for their significant contribution to the super-coarse aerosols present in agro-forest atmospheres. Research conducted on subtropical Okinawa Island in the western North Pacific Rim highlights the diverse composition of PBAPs, which primarily consist of vegetative residues, pollen, and fungal spores rich in saccharides and sugar alcohols (Zhu et al. 2015). These findings underscore the importance of understanding the role of PBAPs in atmospheric processes. PBAPs are of international concern due to their potential health impacts, including allergies, respiratory diseases, and even cancer (Kim et al. 2018). In the Amazon forest biosphere, studies by China et al. (2018) have shown that fungal spores contribute significantly, constituting at least 30% of sodium salt particles during the wet season. Additionally, it is hypothesized that halide-containing fungal spores may play crucial roles in cloud formation processes within the Amazon basin (China et al. 2018).

During the dry season, fine-mode forest aerosols in the Thailand’s deciduous forests are primarily consisted of organic matter from biomass burning smoke, secondary inorganic aerosols, organic matter from other sources, and crustal materials (Oanh et al. 2016). The coarse fraction comprises organic matter from non-biomass burning sources, local inorganic particles, crustal materials, and aged sea salt from distant origins (Oanh et al. 2016). Tsai et al. (2013) reported that significant emissions from forest and agricultural fires, along with soil biota, greatly contribute to episodic PM_10_ pollution events in the Chiang Mai Basin in northern Thailand. Additionally, Punsompong and Chantara (2018) examined coarse aerosol particles in the northern Thailand and confirmed that burning forests in Myanmar, driven by prevailing winds from March to April, is a major source of these particles.

During atmospheric aging, biomass burning aerosols undergo significant transformations influenced by various environmental conditions such as humidity, dryness, darkness, and exposure to UV light (Yazdani et al. 2022). These transformations lead to changes in the chemical composition and properties of both primary and secondary biomass burning aerosols. Interactions between water-soluble organic compounds from biomass burning smoke aerosols and marine aerosols result in complex chemical reactions. This can lead to a decrease in the chloride to sodium ratio of marine aerosols as the organic acid components increase during aerosol aging. The reactions with acids on the surface of marine aerosols can generate HCl, which subsequently evaporates from the particles. Ghorai et al. (2014) found that this HCl evaporation leads to the depletion of chloride and the formation of sodium malonate and sodium glutarate salts.

Chloride aerosols play significant roles in the dynamic properties of aerosols during long-range transport, yet they are often overlooked in atmospheric chemical modeling (Wang et al. 2017). These chloride particles can be internally or externally mixed with primary aerosols, including inorganic salts and organic compounds, altering the hygroscopic and optical characteristics of aerosols and affecting aerosol-cloud interactions and climate forcing potential (Ghorai et al. 2014; Wang et al. 2017). In pristine marine and coastal regions, chloride aerosols may originate from sea salt and internal forest sources, such as soil organisms and plant emissions (Öberg 2003). In inland vegetated areas, they are likely derived from biomass burning activities and saline soils (Öberg 2003). In contrast, industrial urban areas often exhibit chloride aerosols associated with heavy or transitional minerals (Jordan et al. 2015; Wang et al. 2017). Secondary chloride aerosols can also form through various reactions. Primary chlorides can react with both organic and inorganic acids, releasing gaseous HCl, which subsequently reacts with atmospheric NH_3_ or N_2_O_5_ to generate N-containing chloride aerosol particles (Wang et al. 2017). These processes significantly influence the dynamic hygroscopicity and radiative forcing properties of aerosols (Braun et al. 2017), emphasizing the importance of considering chloride aerosols in atmospheric modeling and understanding their implications for environmental and climate dynamics. Additionally, chloride aerosols may play a role in nutrient cycling within forest ecosystems by enhancing the availability of essential nutrients through chemical reactions in the soil and foliage. This can lead to changes in soil pH, salinity, and the availability of other ions, ultimately affecting microbial activity and plant nutrient uptake (Öberg 2003).

Elemental compositions of aerosols are widely used for source identification in various studies, including research conducted at forest sites in Thailand (Oanh et al. 2016). Scanning electron microscopy (SEM) and energy-dispersive X-ray spectrometry (EDS) offer significant advantages for investigating individual coarse-mode aerosol particles by providing both surface morphological characteristics and elemental compositions. Fourier-transform infrared (FTIR) spectroscopy further enhances source identification by revealing functional groups. Gupta et al. (2024a) utilized both SEM/EDS and FTIR to distinguish between biogenic and anthropogenic origins of coarse-mode aerosol particles in the Himalayan region. These techniques can also be applied to studies of geogenic dust, helping to identify crystalline phases and clay mineral types linked to potential sources such as dust storms, rock weathering, demolition, and construction (Gupta et al. 2024b).

In this study, our objective is to comprehensively analyze the morphology and elemental composition of individual aerosol particles across all size ranges within a tropical agro-forest environment, aiming to elucidate their potential sources. Specifically, we narrow our focus to super-coarse carbonaceous aerosol particles containing chloride. Through detailed examination utilizing SEM/EDS and FTIR analyses, we investigate the likely sources of these super-coarse aerosols. The findings from our research may contribute to a deeper understanding of the role of aerosols in Earth’s climate system by shedding light on their influence on radiative balance, cloud formation, and biogeochemical processes. These insights are crucial for informing environmental management strategies and advancing our understanding of the complex interactions between aerosols and the environment.

## Methodology

### Sampling sites and sampling

**Fig. 1:**
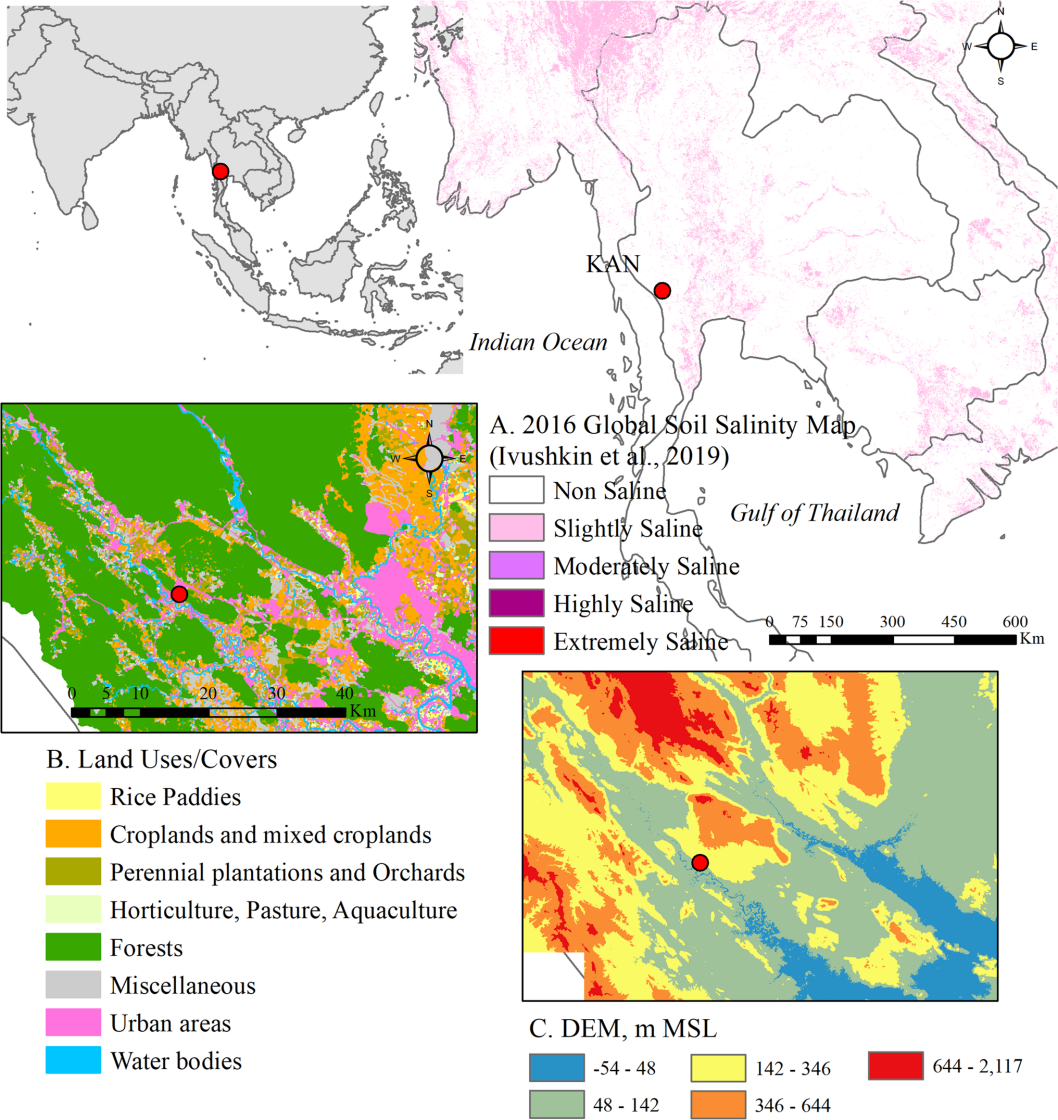
Maps of KAN site overlaid on 2016 global soil salinity map^*^ (**A**), land uses/covers map^**^ (**B**), and Digital Elevation Model (DEM) map^***^ (**C**). Note: ^*^ acquired online from ISRIC – World Soil Information via https://data.isric.org/geonetworadk/srv/api/records/c59d0162-a258-4210-af80-777d7929c512, accessed on 20 April 2024, ^**^ acquired from the Land Development Department, Thailand through Open Government Data of Thailand, https://data.go.th/th/dataset?res_format=SHP, accessed on1 September 2023, ^***^ based on 30 m SRTM DEM, acquired from https://dwtkns.com/srtm30m/, accessed on 18 February 2023

A sampling site at Wimandin Training Center, Mahidol University Kanchanaburi Campus, Sai Yok district, Kanchanaburi (KAN), was selected for this study to represent an agroforestry area (see Fig. 1). The KAN site is situated in an undulating carbonate rock plain, surrounded by mixed deciduous forests and agricultural lands primarily used for cassava, sugarcane, and Para rubber cultivation. The sampling location was within a low-population density educational institution, positioned at 2.5 m above ground level and 162 m above mean sea level.

The sampling sites are located in central Thailand and are influenced by a tropical savanna (AW) climate, according to the Köppen climate classification. Aerosol particles were collected during the dry season from November to January 2020 to 2022 when prevailing northeasterly winds blew from the Chinese Mainland. According to long-term records, minimum and maximum daily temperatures for Kanchanaburi during November to January ranged from 12.8–18.9 °C and 34.0–35.8 °C, respectively (National Statistical Office 2023). Air pollution could be exacerbated during this season due to prolonged high-pressure systems. In the dry season at KAN, deciduous trees begin to defoliate. Local residents typically initiate fires in the forests for easier hunting and harvesting of forest produce. Agricultural biomass burning also intensifies to facilitate crop harvesting and land preparation. Detailed meteorological conditions and PM_2.5_ levels during the sampling periods are provided in Table 1.

**Table 1:** Locations, meteorology, and PM_2.5_ concentrations observed at the two sampling sites

Site	Description	Sampling periods		Hourly average during the course of sampling			
			Air temperature (°C)	Relative humidity (%)	Wind speed (m s^−1^)	Prevailing wind direction	PM_2.5_, 24-h average (μg m^−3^)
Mahidol University, Sai Yok district, Kanchanaburi (KAN)	Agro-forest site, 14°07′4.7″N, 99°09′7.9″E, at 2.5 m above the ground	4–6 January 2022	23.8	72	1.24	NE to SE	18.6–19.4
		7–9 January 2022	24.0	67	1.01	SE to SW	27.2–53.5
		9–12 January 2022	23.8	63	1.08	SE to SW	37.2–53.5
		13–15 January 2022	23.8	70	1.13	E to S	36.2–55.7
		16–18 January 2022	24.2	63	1.23	E, SW	39.9–68.3
National Ambient Air Quality Standard, 24-h average							37.5

Aerosol particles were collected using a DustTrak DRX Aerosol Monitor 8533 with a flow rate of approximately 3 l per minute for a duration ranging from 10 to 24 h. The aerosol particles were deposited on a silver membrane filter (37 mm diameter, 0.8 µm pore size, SKC Inc.). Concurrently with the sampling, fine aerosol concentration (PM_2.5_) was monitored using the DustTrak, and meteorological data were recorded using a mobile weather station (WS-GP1, Delta-T Devices). The samples were stored in a desiccator for approximately 1 month before analysis.

### Sample analyses

#### SEM and EDS

One-fourth of each filter sample was cut and underwent platinum coating before examination using SEM (JEOL, model: JSM-IT200) coupled with EDS (JEOL MP-04110BED). In this analysis, the SEM operates at an electron energy of 20 keV. The EDS subsequently measures the energy of X-rays emitted when the electron beam interacts with the surface of the aerosol particles. The full width at half maximum (FWHM) for the EDS has been maintained within a threshold of 133 eV to ensure optimal equipment performance, yielding reliable and consistent results. To enhance the accuracy of quantitative elemental analysis, ZAF (atomic number, absorption, fluorescence) corrections were applied. SEM observations provided insights into the surface morphology, shape, and size of the aerosol particles, while EDS facilitated the determination of their elemental compositions.

The SEM/EDS analyses were conducted on 31 to 45 particles for each filter sample. Size (maximum cross-sectional length) and shapes were observed from SEM images and documented. Seven types of shapes were classified for ease of recording and analysis: (1) sphere/spheroid, (2) irregular, (3) columnar, (4) rectangular/cubic, (5) fractal (chain-like) aggregate, (6) biogenic, and (7) sheet-like structures. Due to the inability of SEM to clearly distinguish rim and surface morphology in particles smaller than 0.8 µm, these finer particles were excluded from the individual particle analysis.

#### FTIR spectroscopy

Another one-fourth of the silver membrane filter samples was used for functional group analysis using FTIR spectroscopy (Nicolet™ iN10 Infrared Microscope, ThermoFisher Scientific). Fifty-five aerosol particles exhibiting super-coarse size (> 10 µm) and dark colors on the filter samples were selected for this analysis. The aerosol particles analyzed by FTIR were derived from the same filter samples used for SEM/EDS analysis; however, they are not the same super-coarse particles reported in the SEM/EDS results. Prior to analysis, the samples were dried in a silica gel container. FTIR measurements were conducted with a DTGS detector, employing acquired spectra in diffuse reflectance mode spanning from 600 to 4000 cm^−1^ with a resolution of 4 cm^−1^. An aperture size of 10 µm × 10 µm was set, and 16 scans were performed for each sample.

To enhance accuracy for FTIR analysis, a background ambient spectrum was characterized and automatically subtracted from each aerosol spectrum using Omnic software. Additionally, filter spectra were analyzed on a clean filter and manually subtracted from the sample results. Liquid nitrogen was utilized to minimize interference and improve the signal-to-noise ratio of the FTIR spectra.

Functional group identification relied on the IR absorbance peaks, referenced from previous publications and summarized in Table 2. Spectra within the range of 2000–2500 cm^−1^ were excluded from this analysis due to CO_2_ absorption band interference.

**Table 2:** FTIR absorption peaks used in quantification of aerosol function groups

Functional or structure groups	Wavenumber (cm^−1^)	References
Clay minerals (anhydrate), including illite, kaolinite, montmorillonite	844–938, 3620–3703	Gupta et al. (2024b), Jozanikohan and Abarghooei (2022)
O–H, hydroxyl group	3100–3550	Gilardoni et al. (2009), Shankar et al. (2022)
C = O, carbonyl group	1640–1850	Gilardoni et al. (2009), Maria et al. (2002), Polidori et al. (2008)
–C–H stretching, alkanes	2800–3000	Gilardoni et al. (2009)
= C–H stretching, alkenes	2900–3100	Gilardoni et al. (2009), Polidori et al. (2008)
C–C–H, aliphatic carbon	1370–1480	Polidori et al. (2008)
NO_3_^−^, nitrate ions	1335–1410	Polidori et al. (2008); Shankar et al. (2022)
− NO_2_, nitro group (aromatic)	1525–1535	Polidori et al. (2008)
C–O–S, organosulfate group	876	Corrigan et al. (2013), Gilardoni et al. (2009)
Levoglucosan	860–1050	Yazdani et al. (2022), Zhu et al. (2015),
Black carbon	500–670	Shankar et al. (2022)

It is important to note that both the electron beam from SEM and the IR beam from FTIR may not penetrate the inner regions of all aerosol particles, especially those that are thick and contain carbonaceous compounds, which exhibit strong IR absorption.

### Quality assurance and quality control

A filter blank was prepared alongside filter samples for each sampling batch. Both the blank and filter samples were weighed using a microbalance (Sartorius Model: MSA36S-000-DH) and stored in the same clean, desiccated container prior to sampling. After sampling, both the blank and samples were desiccated and reweighed. In this study, the pre- and post-weights of the blank filters were controlled to within ± 100 µg to ensure proper handling. All analytical instruments used in this study are calibrated and maintained annually by certified service providers.

### Data collection and analyses

#### Air back trajectory analysis

A 5-day air back trajectory analysis was conducted using the Hybrid Single Particle Lagrangian Integrated Trajectory Model (HYSPLIT) to depict potential aerosol sources at the sampling site, KAN. The model was interactively operated via NOAA’s Air Resources Laboratory (ARL) website, accessible at http://ready.arl.noaa.gov/HYSPLIT.php. The frequency grid resolution was set at 1° × 1° degrees. Meteorological data utilized for the analysis were sourced from the Global Data Assimilation System (GDAS) within the archive reanalysis project, a collaboration between the National Centers for Environmental Prediction (NCEP) and the National Center for Atmospheric Research (NCAR).

#### Positive Matrix Factorization

Positive Matrix Factorization (PMF) was employed to discern the predominant features of individual aerosol particles based on their elemental mass percentages, with standard deviations ranging from 0.03 to 1.92%, depending on the mass fraction and element. The factorization process utilized an iterative algorithm, commencing with random initial values for nonnegative factor loadings (H) and factor scores (W). Subsequently, W and H were estimated by minimizing the root-mean-square residual between the observation and its lower-rank approximation (W×H). Elevated factor loadings on specific heavy metals imply their significant contribution to the factor. The identified four factors likely encapsulate essential attributes of the dry-season aerosol particles at the KAN site, with the root-mean-square residuals accounting for 1.614.

## Results and discussion

### SEM/EDS morphology and elemental compositions of the individual aerosol particles

**Fig. 2:**
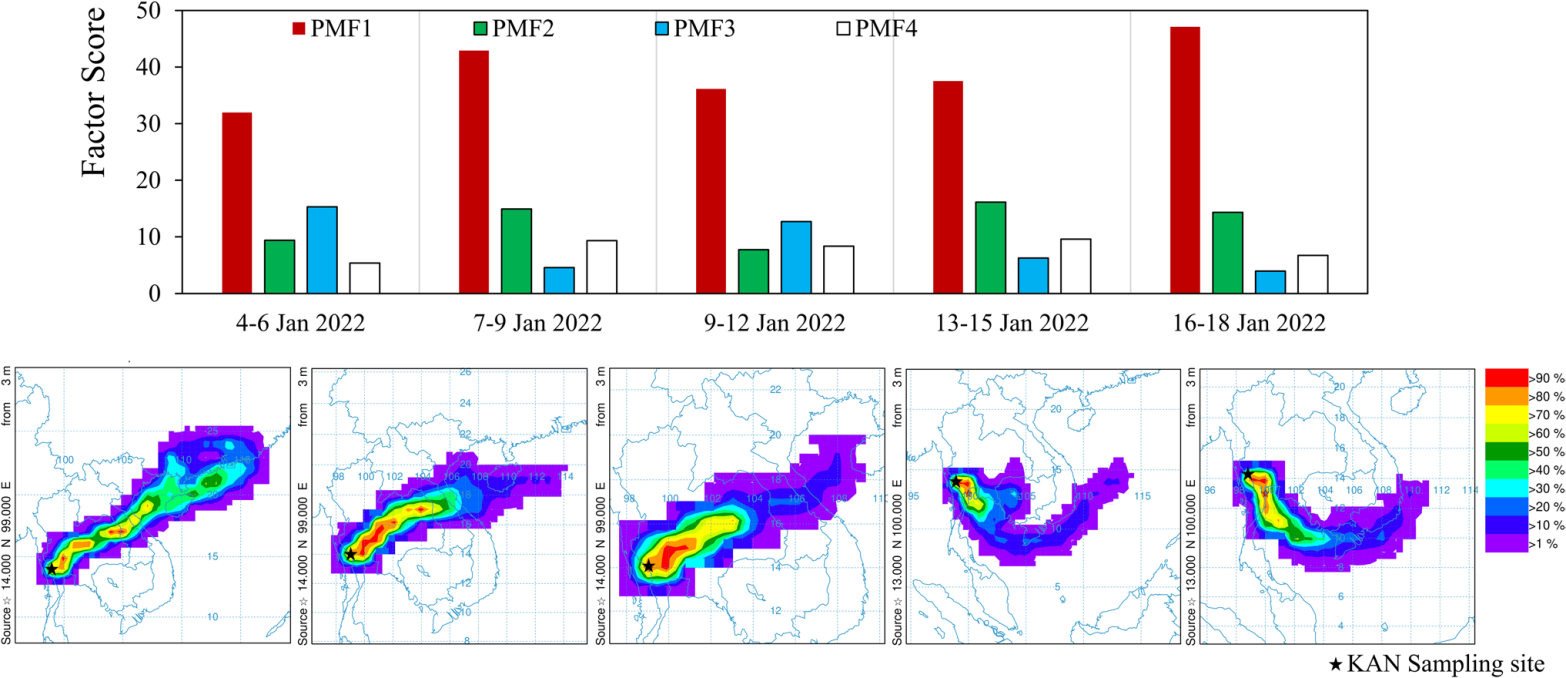
The factor scores for PMF1 to PMF4 (as listed in Table 2) during the sampling periods, alongside their temporally corresponding 5-day air back trajectories at the KAN site

Based on the 5-day air back trajectories during the dry season depicted in Fig. 2, KAN received air masses from both the northeast and central Thailand, carried by northeasterly (NE) winds, as well as from the Gulf of Thailand, driven by southeasterly (SE) winds. Additionally, wind direction observations from the local KAN meteorological station (refer to Table 1) indicated prevailing southwesterly and SE winds, influenced by the geographical landscape.

**Fig. 3:**
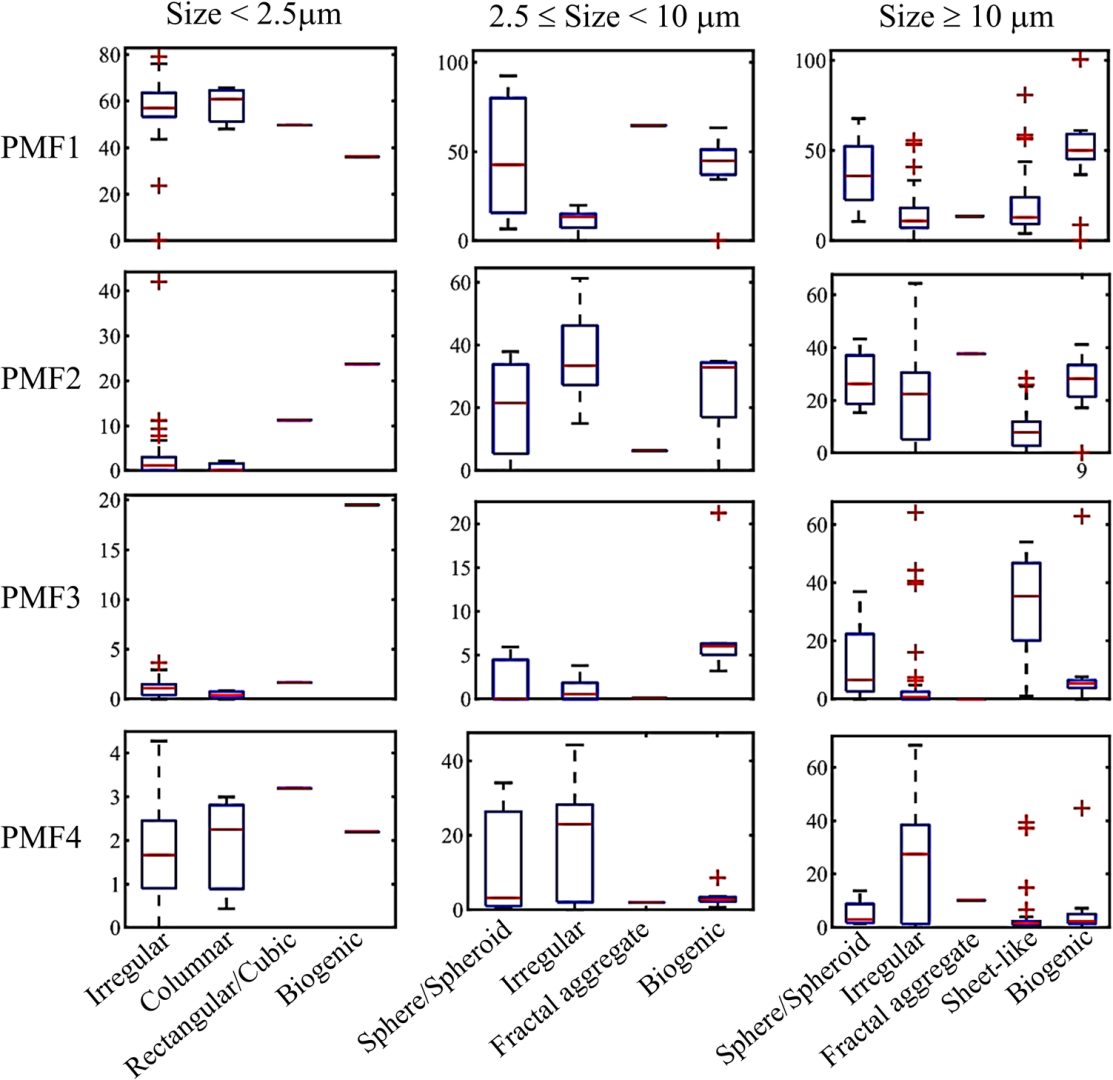
Factor scores of the PMF1 to PMF4 against aerosol shapes and size ranges

Four PMF derived from the elemental compositions of the aerosol particles were attributed to compositions from the agriculture-forest site (KAN). The PMF loadings for elemental compositions of the aerosol particles are presented in Table 3, while the PMF scores for various aerosol sizes and shapes are depicted in Fig. 3. Based on these findings, the key aerosol characteristics and sources can be assessed as follows.

**Table 3:** The factor loadings on the elements of individual aerosol particles sampled at KAN

	PMF1	PMF2	PMF3	PMF4
C	**0.998**	-	**0.712**	**0.126**
O	0.060	**0.979**	**0.596**	**0.836**
N	-	-	**0.368**	-
K	-	0.008	0.010	0.017
Ni	0.011	-	-	-
Zn	0.009	-	-	-
Ca	-	**0.154**	-	-
Mn	0.005		-	-
Fe	-	0.039	-	0.048
As	0.011	-	-	0.012
Si	-	-	-	**0.524**
Cl	-	-	0.020	-
Mg	-	-	0.005	0.032
Al	-	**0.129**	-	0.077
P	0.006	-	-	0.006
S	0.012	-	0.011	-
Na	-	-	0.036	-
Cu	0.008	-	-	-
Pb	0.025	-	-	0.021

“-” means the loading less than 0.05; Bold number indicates the elements with high factor loading onto the PMF

As indicated by the factor loading in Table 3, PMF1 primarily comprised black carbon (C), with minor fractions of oxygen (O), lead (Pb), sulfur (S), nickel (Ni), and arsenic (As), respectively. PMF1 scores predominated throughout all sampling periods in the dry season. Aerosol particles exhibiting high PMF1 scores were found across fine (< 2.5 µm) to super-coarse sizes (> 10 µm), with fine aerosols likely displaying irregular, columnar, or rectangular (or cubic) shapes, while coarser sizes tended to be biogenic (refer to Fig. 3). Based on the key tracer elements Pb, Ni, As, and S, coal combustion may contribute to the characteristics of PMF1, similar to observations made in the UK (Vincent and Passant 2006) and India (Pant and Harrison 2012).

PMF2 aerosol particles were identified as mineral dusts, calcium (Ca)-aluminium (Al)-rich with moderate iron (Fe), likely exhibiting coarse sizes (≥ 2.5 µm) and either irregular or spherical/spheroidal shapes. These particles are presumed to be geogenic, aligning with the predominant limestone geography in KAN. Silicate dust particles, as represented by PMF4 characteristics, were also prominent, often displaying coarse irregular particles composed primarily of O and silicon (Si), moderately C, and minor amounts of Al, Fe, magnesium (Mg), Pb, and potassium (K). Due to the high carbon content in PMF4 and the presence of K, it is hypothesized that the silicate dust particles may have originated in conjunction with biomass burning smoke (Pant and Harrison 2012). Furthermore, at high PMF4 scores (> 10), the PMF4 characteristics in the dust were inversely proportional to PMF2, suggesting that both PMF2 and PMF4 characteristics were not triggered by the same source.

**Fig. 4:**
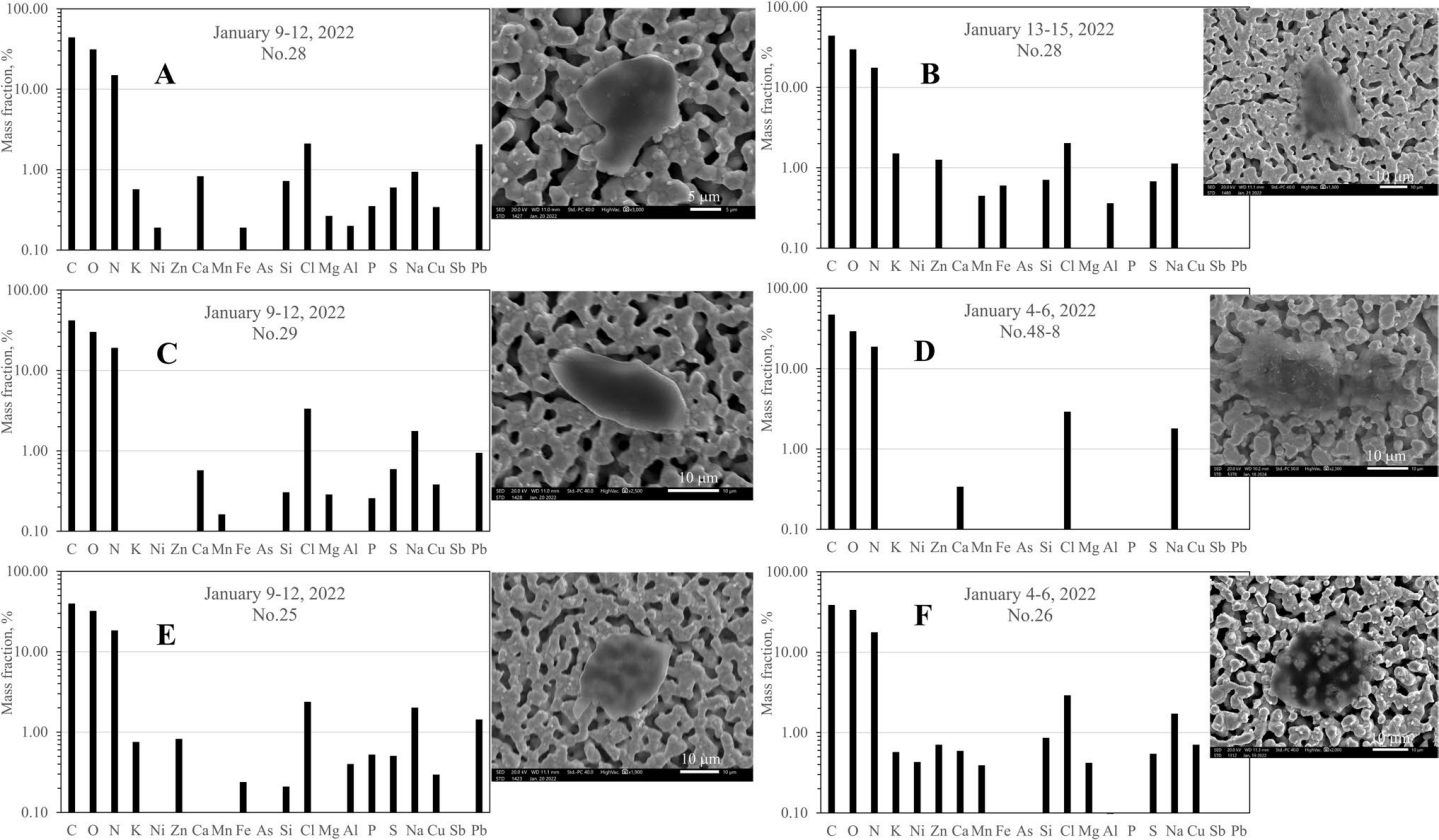
SEM images and EDS elemental profiles for aerosol particles collected at KAN exhibiting strong PMF3 characteristics

PMF3 aerosol particles were identified as organic, C-, O-, nitrogen (N)-rich, with trace amounts of sodium (Na), chlorine (Cl), S, and K (refer to Table 3). These carbonaceous particles exhibited unique morphological forms of super-coarse sheet-like structures (refer to Fig. 4). The sources of these PMF3 particles remain uncertain; however, potential sources have been discussed in the “PMF3 super-coarse carbonaceous aerosol particles containing chloride” section.

### FTIR compound functional groups of the super-coarse aerosol particles

To differentiate primary bioaerosols from biomass burning smoke particles and inorganic salts, we investigated the functional groups of individual particles. We hypothesized that bioaerosols primarily contain sugar alcohols, characterized by functional groups such as hydroxyl group (3100–3550 cm^−1^), carboxyl group (1640–1850 cm^−1^), and long hydrocarbon chains—alkane (2800–3000 cm^−1^) or alkene (2900–3000 cm^−1^). In contrast, carbonaceous aerosol particles from pollution sources should exhibit the presence of black carbon (500–670 cm^−1^) and levoglucosan (860–1050 cm^−1^) indicating combustion origins.

**Fig. 5:**
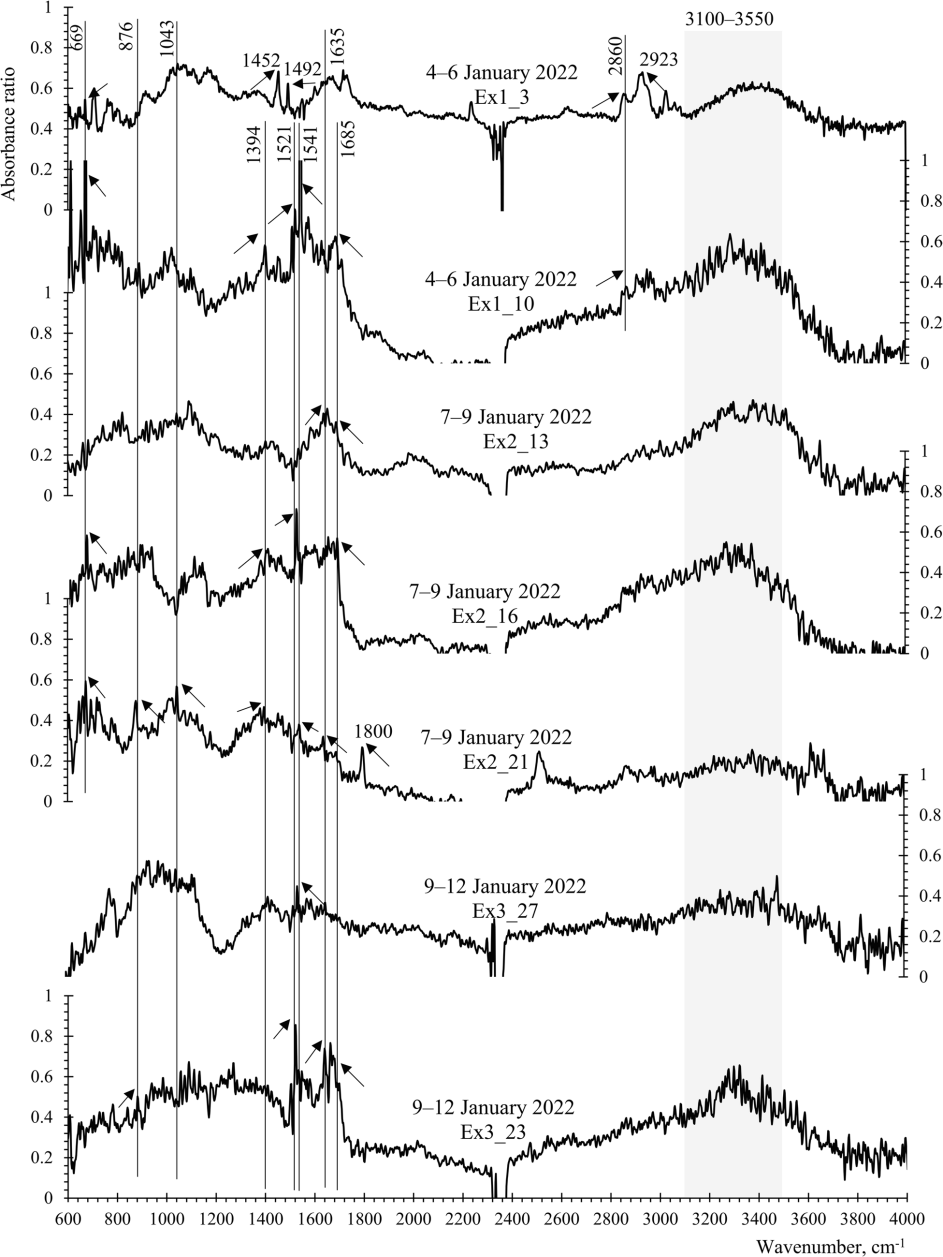
Absorption of the electromagnetic spectrum against wavenumber for the aerosol particles collected from January 4 to 12, 2022, during prevailing northeasterly winds

**Fig. 6:**
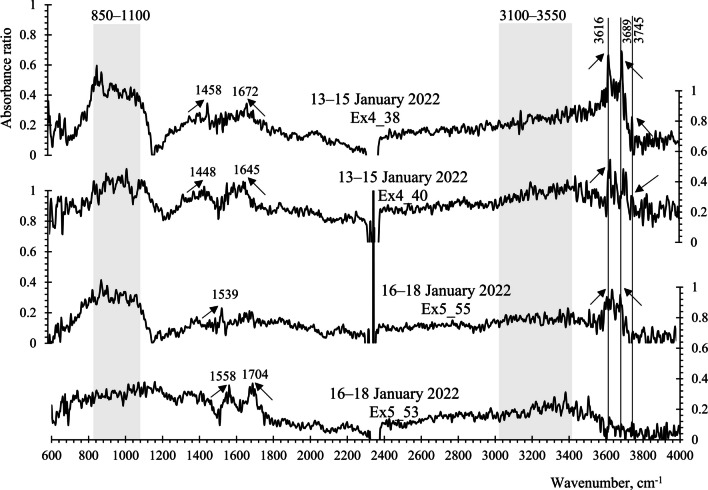
Absorption of the electromagnetic spectrum against wavenumber for the aerosol particles collected from January 13 to 18, 2022, during prevailing southeasterly winds

The analysis of super-coarse (> 10 μm) aerosol particles with dark color using FTIR spectroscopy revealed compounds with major functional groups. The variations in functional or structural groups of the aerosol particles associated with NE and SE winds are illustrated in Figs. 5 and 6 and summarized in Table 4. These include.

1. Hydroxyl group (O–H), including water, alcohols, and polysaccharides. Super-coarse aerosol particles (Ex1_3, Ex1_10, Ex2_13, 2_16, 3_23) associated with NE winds exhibited absorption corresponding to broad O–H stretching bands of 3100–3550 cm^−1^, suggesting hygroscopic properties (Gilardoni et al. 2009; Shankar et al. 2022). Aerosol particles containing hydroxyl groups likely originate from biogenic sources, which were bioaerosols or biomass burning (Popovicheva et al. 2020). However, this absorption band was less likely found in dry aerosol particles associated with SE winds (see Fig. 6, no major O–H absorption band).
2. Carbonyl group (C = O), including aldehydes, ketones, carboxylic acids, and amides. Strong absorption at about 1645–1704 cm^−1^ for the SE-winds-influenced aerosol particles (Ex4_38, Ex4_40, Ex5_53) and 1635 and 1685 cm^−1^ for the NE-winds aerosol particles (Ex1_10, Ex2_13, Ex2_16, Ex2_21, Ex3_23) responded to compounds in the carbonyl group (Gilardoni et al. 2009; Maria et al. 2002; Polidori et al. 2008), indicating possible sources from fossil fuel combustion or biomass burning. Aerosol particles containing carboxyl groups may originate from either fossil fuel combustion (such as diesel and gasoline) or biomass burning (including both flaming and smoldering phases) (Popovicheva et al. 2020). Similar findings were reported during the sugarcane burning season in southern Brazil, where higher fluxes of carboxylic acids were observed (Da Rocha et al. 2005).
3. Aliphatic compounds, including saturated alkanes and unsaturated alkenes. These compounds exhibited strong absorption bands of 1370–1480 cm^−1^ and 2800–3000 cm^−1^ (Gilardoni et al. 2009; Polidori et al. 2008). Both aliphatic carbons and carbonyl compounds were observed together in the SE-winds aerosol particles (Ex4_38, 4_40, 5_53), suggesting same origins or possible oxidation of aliphatic carbons to carbonyl compounds during aging (Gilardoni et al. 2009).
4. Nitrate ions (NO_3_^−^), which absorb at 1335–1410 cm^−1^, and nitroaromatic compounds, which absorb at 1525–1535 cm^−1^ (Polidori et al. 2008; Shankar et al. 2022), were frequently detected in the aerosol particles induced by NE winds (Ex1_10, Ex2_16, Ex2_21, Ex3_23, Ex3_27), with absorption spectra around 1394 cm^−1^ and 1521, 1541 cm^−1^, respectively. These compounds are commonly found in urban dust (Polidori et al. 2008) and in smoke particles from biomass burning, including both flaming and smoldering (Popovicheva et al. 2020).
5. Byproducts of biomass burning include the organosulfate group, levoglucosan, and black carbon aerosols. They exhibit vibrations at 876 cm^−1^, 860–1050 cm^−1^, and 500–670 cm^−1^, respectively (Corrigan et al. 2013; Gilardoni et al. 2009; Shankar et al. 2022; Yazdani et al. 2022; Zhu et al. 2015). Both organosulfate and levoglucosan were observed in some aerosol particles associated with NE winds (Ex2_21 and Ex3_23), showing absorptions at ~ 876 cm^−1^ and ~ 1043 cm^−1^, respectively. The presence of these compounds suggests that the NE winds likely carried biomass burning smoke and its associated super-coarse aerosol particles to the KAN site. Additionally, both types of aerosol particles contained nitroaromatic compounds. A similar aerosol composition has been found in urban environments influenced by biomass burning in Ljubljana, Slovenia (Frka et al. 2022). In addition, the strong absorption peak at ~ 669 cm^−1^ for the NE-winds aerosol particles (Ex1_10, 2_16, 2_21) suggests the presence of black carbon, confirming the burning origins.
6. Anhydrous clay minerals exhibit distinctive absorption peaks at 840–1100 cm^−1^ and 3616–3745 cm^−1^, as reported by Jozanikohan and Abarghooei (2022). These minerals were notably prevalent during periods of prevailing SE winds (see Fig. 6).

**Table 4:** Major functional or structural groups and absorption spectra of the dust particles linked to NE and SE winds

Prevailing NE winds, 4–12 January 2022		Prevailing SE winds, 13–18 January 2022	
Functional groups	Wavenumber, cm^−1^	Functional groups	Wavenumber, cm^−1^
Black carbon	669	C–C–H, Aliphatic Carbon	1448–1558
C–O–S, organosulfate group	876	C = O, Carbonyl group	1645–1704
Levoglucosan	< 1043	Clay minerals	840–1100, 3616–3745
NO_3_^−^, nitrate ions	1394		
C–C–H, aliphatic carbon	1452, 1492		
− NO_2_, nitro group (aromatic)	1521, 1541		
C = O, carbonyl group	1645–1704		
–C–H stretching, alkanes	2860, 2960		
O–H, hydroxyl group	3100–3550		

From the findings, we propose that super-coarse aerosols carried by prevailing NE winds may be affected by biomass burning, while those influenced by SE winds are likely sourced from soils and activities unrelated directly to local biomass burnings.

### PMF3 super-coarse carbonaceous aerosol particles containing chloride

In the remote mountainous area of KAN, we discovered super-coarse aerosol particles rich in C-O-N content, displaying a sheet-like structure and likely containing halide minerals such as NaCl or KCl. The morphology and chemical compositions of these aerosol particles are illustrated in Fig. 4. Two hypotheses have been proposed to explain the sources of these aerosol particles: they could be a mixture of biomass burning smoke particles with inorganic salts, or they may originate from halophilic fungal spores.

#### Mixed biomass burning smoke particles with inorganic salts?

We speculated that the aerosol particles could be aged biomass burning smoke particles mixed with other organic compounds, developing hygroscopic growth induced by inorganic salts during long-range transportation. Typically, organic aerosol particles from biomass burning smoke, containing a sum of C, O, and/or N of more than 90%, appear rounded or with irregular shapes (Fu et al. 2012). The aerosols could be coated by an N- or S-rich rim, suggesting oxidation by NO_x_ or SO_2_ in the atmosphere (Fu et al. 2012; W. Li and Shao 2010). Li et al. (2003) reported an abundance of organic particles and soluble salts in biomass burning smoke plumes in South Africa, discussing the hygroscopic properties of these aerosol particles. Zhang et al. (2020) observed similar evolution of organic aerosol particles during haze episodes in both urban and rural sites in northeast China. Geng et al. (2009) also found an abundance of C-, N-, and O-rich droplets in Asian dust aerosol samples collected in the marine atmosphere between South Korea and Beijing, China. Dang et al. (2022) elaborated on aged biomass burning and marine aerosols over the SE Atlantic Ocean, discussing heterogeneous reactions involved with aged biomass burning smoke aerosols, resulting in higher fractions of volatile organic compounds, Na, and N. The high fraction of N in these PMF3 organic aerosol particles could be from reactions with HNO_3_, NH_3_, and NH_4_NO_3_ in the smoke plumes (Dang et al. 2022; Geng et al. 2009). Furthermore, the relative humidity of 60–70% during the sampling periods could facilitate the hygroscopic growth of these aerosols (Dang et al. 2022).

The FTIR results, shown in Table 4, indicate that nitrogen found in the super-coarse KAN aerosol particles was likely in the form of nitroaromatics and nitrate ions, induced by prevailing NE winds from the Asian Mainland, not likely by SE winds. During this period, biomass burning dusts had been dominant, as implied by their compositions of organosulfate, levoglucosan, and black carbon (Table 4).

The super-coarse sheet-like structure and transparency of many of these PMF3 organic aerosol particles (see Fig. 4) could be attributed to dry droplets of condensates from mixed biomass burning smokes. The halide minerals exhibited high hygroscopicity, enhancing water uptake and growth of the condensate droplets. Potassium chloride (KCl) is commonly used as a proxy for biomass burning smoke aerosols since it was consistently found in both fresh and aged biomass burning smoke particles (Diapouli et al. 2014; Li et al. 2003; Wang et al. 2017).

Nevertheless, the KAN organic chloride particles represented by PMF3 often contained NaCl. As shown in Table 5, the mean and median molar ratios of Na and Cl for PMF3 particles (PMF3 score > 2.92) were close to or slightly higher than unity, whereas the molar ratios of K and Cl were markedly lower. The sources of NaCl in the PMF3 organic aerosol particles remain uncertain, whether from sea spray or crustal origin from saline soils. The sampling site is distant (140 km) from the shorelines. Sea salt could be long-range transported from either the Pacific or Indian Oceans, induced by the monsoon climate systems. Reactions of oxides of nitrogen with sea salt particles result in volatilization of Cl mainly attributed to chloride depletion, as observed on the US east coast and the Pacific Ocean (Newberg et al. 2005; Pio and Lopes 1998; Zhao and Gao 2008). In the case of KAN, aerosol particles with high PMF3 characteristics exhibited mean Na-to-Cl molar ratios of 1.33 and 1.21 for those PMF3 of 2.92–34.5 and > 34.5, respectively. Ratios higher than unity could result from chlorine volatilization during long-range transport.

**Table 5:** Molar ratios of Ca to Na, Na to Cl, and K to Cl for individual particles with different PMF3 characteristics

PMF3	Number observations	Mean ± standard error			Median		
		Ca/Na	Na/Cl	K/Cl	Ca/Na	Na/Cl	K/Cl
0–0.36	21	6.31 ± 2.40	0.90 ± 0.23	1.48 ± 0.75	0	0.5	0
0.36–1.27	21	0.13 ± 0.08	0.33 ± 0.20	0.24 ± 0.15	0	0.06	0
1.27–2.92	20	0.66 ± 0.35	0.19 ± 0.08	0.21 ± 0.13	0	0.06	0
2.92–34.50	21	0.58 ± 0.34	1.33 ± 0.36	0.73 ± 0.30	0.18	1.07	0.23
> 34.50	21	0.20 ± 0.05	1.21 ± 0.10	0.24 ± 0.05	0.11	1.17	0.20

Another possible source of NaCl is saline soils suspended during biomass burnings (Da Rocha et al. 2005). Table 5 also shows comparatively higher Ca to Na molar ratios than seawater (0.114 (Jordan et al. 2015)), implying a crustal origin. As seen in Fig. 2, the saline soil particles could be transported from the Central Plain, where salt-affected soils have developed on marine sediments (Arunin and Pongwichian 2015). The contribution of saline soils could be notable during prevailing SE winds from the Gulf of Thailand, from January 13 to 18, 2022. The results of the FTIR analysis, shown in Table 4, revealed that the SE-wind-influenced aerosol particles should be clay minerals mixed with organic compounds, such as aliphatic carbons and carbonyl compounds.

#### Halophilic fungal spores?

Additionally, a new discovery by China et al. (2018) suggests that chloride aerosol particles may contain sodium salt derived from halophilic fungal spores, including their ruptured forms. The study reported that dominant fungal spores containing sodium salt were found in the Amazon forest, which has a climate similar to Thailand. Previous reports on these fungal spores in the atmosphere have been limited; possibly, they were misinterpreted as sea salt (China et al. 2018). There are striking similarities between the Amazonian fungal spores and the PMF3 KAN coarse carbonaceous aerosols. The fungal spores discovered in the Amazon forest were of super-coarse sizes, rich in C-O-N with significant NaCl content, and exhibited hygroscopic properties (China et al. 2018). Their morphologies were typically flaky and spheroid, potentially indicating ruptured spores (China et al. 2018). However, surface ornamentation, a characteristic feature of fungal spores in general, could not be identified on these spores, leading to uncertainty in concluding the source of these PMF3 particles.

**Fig. 7:**
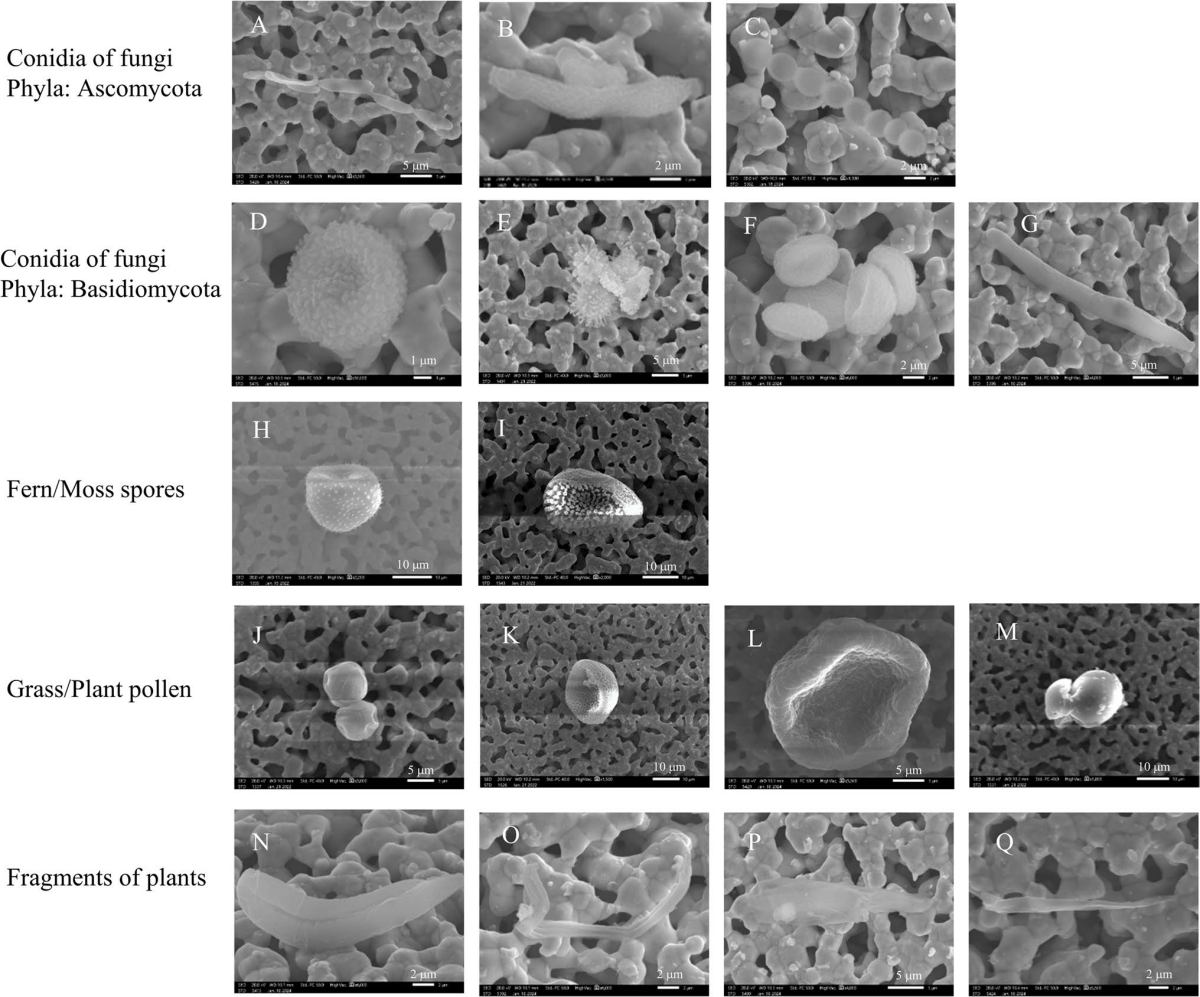
The surface morphology of the KAN bioaerosols, exhibiting strong PMF1 characteristics. **A** to **C** depict conidia of fungi from the phylum Ascomycota, featuring specialized reproductive structures called asci, which contain sexual spores known as ascospores. Some of these ascospores, such as those from *Cladosporium* shown in **A** and **B**, and *Aspergillus* or *Penicillium* in **C**, are potentially asthma-inducing. **D** to **G** show conidia of fungi from the phylum Basidiomycota, each displaying distinct surface ornamentation—baculate (7.1 μm size), echinate (7.7 μm size), striate (6.2 μm size), and regulate (22 μm size). The fungi in this phylum include mushrooms and those causing diseases in various crop plants, such as rusts and smut fungi. **H** to **M** illustrate fern and moss spores, and plant pollen found in KAN. The spores are larger in size (> 10 μm) than those of fungal cells, except for the plant pollen in J, which measures about 8 μm and share morphology similar to *Mimosa* (Leguminosae) pollens. **H** exhibits echinate-type surface ornamentation and is likely a moss spore. Additionally, fragments of plants were also found, as shown in **M** to **P**

In this study, typical fungal spores were frequently categorized as PMF1 super-coarse aerosol particles (see Fig. 3), easily recognizable by their surface morphologies. Figure 7 illustrates the surface morphologies of the bioaerosols observed in the KAN ambient air during the dry season, including fungal spores, fern and moss spores, plant pollens, and fragments of plants probably emitted from biomass burning.

**Fig. 8:**
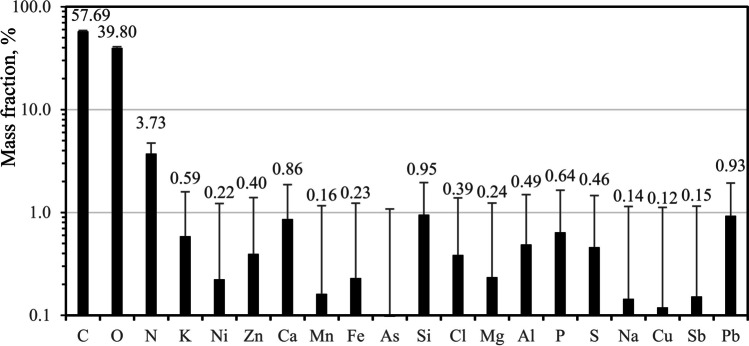
Mean (column and number) and standard deviation (error bar) of elemental compositions of the PMF1 bioaerosols in KAN

Figure 8 presents the mean elemental levels and their standard deviations determined by energy dispersive spectroscopy (EDS) analysis of the bioaerosol particles. The observed elemental compositions reveal an average Cl-to-Na molar ratio of approximately 1.4. This ratio appears higher than anticipated (0.7 ± 0.1 (Ghorai et al. 2014)), suggesting that factors other than halophilic fungi may contribute to the composition of the bioaerosols in this context. Additional research is needed to verify the presence of halophilic fungal spores in the KAN agro-forest mountainous area, situated beyond the Amazon rainforest. This study should also include detailed examinations of the spores’ surface morphology, elemental composition, and seasonal variations in the atmosphere.

### Recommendations for further studies

In this study, we presented evidence to elucidate potential sources of super-coarse carbonaceous aerosol particles containing chloride in a tropical savanna climate at an agro-forest site in Thailand. Our findings suggest the possible presence of mixed biomass burning smoke particles with inorganic salts or halophilic fungal spores, although confirmation is pending. The proposed hypotheses about the origins of these aerosol particles pave the way for further investigation into the roles of biomass burning and halophilic fungi in aerosol formation. This could enhance our understanding of biogeochemical cycles and their impact on atmospheric processes, which is vital for understanding the interconnectedness of Earth’s systems. Such insights have important implications for environmental health, climate change mitigation, and sustainable resource management.

To address this aspect, we recommend further monitoring of seasonal variations in the super-coarse aerosol particles and their correlations with meteorological parameters. Sampling the aerosol particles with size-segregating samplers, such as cascade impactors or cyclone separators, can help minimize interference from submicron aerosol particles during analysis. These observations could enhance our understanding of how the aerosol particles relate to agro-forest activities. Additionally, the use of transmission electron microscopy (TEM) techniques could provide visualizations of the particles’ structures, distribution, and interactions among different elements. Moreover, TEM could offer detailed visualizations of organelles, membranes, and cell walls of fungi, facilitating the confirmation of the presence of halophilic fungal spores.

## Conclusion

The research study aimed to investigate the sources and characteristics of super-coarse carbonaceous aerosol particles containing chloride in a tropical savanna climate. Aerosol samples were collected from an agro-forest site in Thailand (KAN) during the dry season and analyzed using SEM, EDS, and FTIR spectroscopy.

## Key findings include

- PMF analysis of aerosol elemental compositions identified four distinct factors: black carbon (PMF1), mineral dust (PMF2), silicate dust (PMF4), and super-coarse sheet-like organic particles containing halide minerals (PMF3). Each factor exhibits unique morphologies and sizes.
- FTIR analysis of the super-coarse dark aerosol particles revealed functional groups such as hydroxyls, carbonyls, and aliphatic compounds, indicating potential sources like biomass burning and fossil fuel combustion. Aerosols from southeast winds are linked to soil and mineral origins, while those from northeast winds are primarily associated with biomass burning.

Based on these findings, two hypotheses regarding the origin of PMF3 aerosols were proposed:

- Hypothesis 1: Aged biomass burning smoke mixed with organic compounds and inorganic salts, supported by FTIR results and the potential contribution of saline soils to chloride dust during biomass burning events.
- Hypothesis 2: The presence of sodium salts from halophilic fungal spores, which requires further investigation.

The study recommends ongoing monitoring and correlation with meteorological parameters to enhance understanding of aerosol behaviors related to agro-forest activities. It also suggests using TEM for detailed visualization and source confirmation. These methodologies aim to improve our understanding of aerosol dynamics in tropical savanna climates, informing environmental management strategies and climate research.

## Data Availability

The datasets generated during and/or analyzed during the current study are available from the authors upon request.
